# Modeling the Ultrasonic Micro-Injection Molding Process Using the Buckingham Pi Theorem

**DOI:** 10.3390/polym15183779

**Published:** 2023-09-15

**Authors:** Marco Salazar-Meza, Oscar Martínez-Romero, José Emiliano Reséndiz-Hernández, Daniel Olvera-Trejo, Jorge Alfredo Estrada-Díaz, Claudia Angélica Ramírez-Herrera, Alex Elías-Zúñiga

**Affiliations:** Institute of Advanced Materials for Sustainable Manufacturing, Tecnologico de Monterrey, Ave. Eugenio Garza Sada 2501, Monterrey 64849, NL, Mexico; a00398431@itesm.mx (M.S.-M.); emi_jose.18@hotmail.com (J.E.R.-H.); daniel.olvera.trejo@tec.mx (D.O.-T.); a01112513@itesm.mx (J.A.E.-D.); claudia.ramirezh@tec.mx (C.A.R.-H.)

**Keywords:** ultrasound, ultrasonic micro-injection, dimensional analysis, polypropylene, Buckingham Pi theorem

## Abstract

Dimensional analysis through the Buckingham Pi theorem was confirmed as an efficient mathematical tool to model the otherwise non-linear high order ultrasonic micro-injection molding process (UMIM). Several combinations of processing conditions were evaluated to obtain experimental measurements and validate the derived equations. UMIM processing parameters, output variable energy consumption, and final specimen’s Young modulus were arranged in dimensionless groups and formulated as functional relationships, which lead to dimensionless equations that predict output variables as a function of the user-specified processing parameters and known material properties.

## 1. Introduction

Ultrasonic micro-injection molding (UMIM) has been used by manufacturers as a mean to blend polymeric parts in the process known as ultrasonic welding, but a great effort is still being expended on researching how ultrasonic energy can be implemented to improve the results of conventional techniques, especially to revolutionize the method used by the traditional process. One of those cases is the aforementioned UMIM process, in which traditional heaters and mixers used in conventional injection molding (CIM) are replaced by an ultrasonic horn, known as a sonotrode, to plasticize the material.

**Figure 1:**
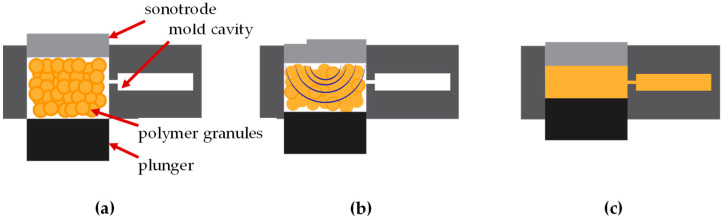
Schematic of the UMIM process. (**a**) The plasticizing chamber is filled with material prior to injection; (**b**) the process starts by inducing longitudinal ultrasonic vibration to the sonotrode, creating a cyclic compression force and heat that melts the pellets; and (**c**) the plunger starts moving upwards, injecting the molten material into the mold cavity.

In the first step of UMIM, the sonotrode is placed over the plasticization chamber where the pellets lie. When it is in position, the sonotrode starts vibrating, the movement increases its temperature [1] while conducting momentum to the pellets, thus creating relative movement among them and the walls of the plasticization chamber, generating interfacial friction [2]; the waves which pass through the polymer lead to shear viscosity [3], generating an internal temperature increase due to viscoelastic heating [4]. Shear viscosity is predominant until the interfaces of the pellets disappear, at which point viscoelastic heating becomes the dominant factor [4,5,6]. Both of these activities, along with cavitation, which occurs in the polymer melt [7,8,9], lead to the full plasticization of the polymer inside the chamber. The sonotrode keeps vibrating and the plunger rises, applying compression force towards the molten material, injecting it through the runners to the mold until it is completely filled, then it proceeds to the packing phase where the specimen is held in place until it is cold. A diagram of the process can be seen in Figure 1.

This UMIM technology has been proven to process polymers like polypropylene (PP) [5,10,11,12,13], biocompatible polymers like polylactide (PLA) [14], engineering polymers such as polyoxymethylene (POM) [4], poly (methyl methacrylate) (PMMA) [1,15], ultra-high molecular weight polyethylene (UHMWPE) [16], polyphenylsulfone (PPSU) [8,17] polyether ether ketone (PEEK) [18], and polybutylene naphthalate (PBN) [19]. In addition, it has demonstrated impressive capabilities to produce composite polymeric materials with fillers like graphite [20], multiwalled carbon nanotubes (MWCNTs) [21], nano-clays [22,23], and even antibacterial drugs [24]. Moreover, a recent study has demonstrated that it is an outstanding method of recycling polymeric materials, since polypropylene recycled through UMIM possesses better mechanical properties than raw polypropylene [10].

Due to the novelty and complexity of the UMIM process, only some articles have investigated the development of theoretical equations and mathematical models to describe its physics. This complexity arises from intricate equations necessary to describe the UMIM process. In the initial stage, the energy required for polymer heating in UMIM comes from viscoelastic deformation and friction provided by the ultrasonic vibrations. When attempting to solve the conservation of energy equation, the different time scales of the thermomechanical coupling problem render the process computationally challenging. Benetar et al. [25] modeled viscoelastic heating when applying sinusoidal deformation and proposed a ratio of stress to strain in vibration conditions. They found that dissipated energy per unit volume in a cycle is proportional to strain amplitudes and stress. Dry friction heating between polymer samples was also modeled by Wu et al. [26] as a function of friction stress and relative sliding velocity. Therefore, understanding the properties of polymer–metal interfacial friction is critical for controlling the manufacturing process of polymer-based products [27].

In the second stage, known as filling, the polymer melt is injected into the mold cavity. Here, the Navier–Stokes equations typically describe fluid mechanics and heat transfer, commonly employed in commercial software simulations. However, applying these simulations to the UMIM process can lead to inaccuracies due to ultrasound-induced viscosity reduction and strong coupling between the heating and filling stages [28].

As expected, the complexity of existing mathematical models has led to a scarcity of simplified models capable of elucidating the nonlinear equations governing the UMIM process [1,4,26,29,30]. There is a notable literature gap when developing an analytical model that captures the physical significance of the various stages of the UMIM process avoiding the intricacies associated with nonlinear mathematical expressions. Although efforts have been made to characterize the UMIM process through extensive in-line monitoring, it can be expensive and challenging, particularly depending on the part design [31]. One promising avenue involves the use of dimensionless groups, which can be defined using the Buckingham Pi theorem [32,33,34].

Dimensionless groups arose in fluid mechanics to provide information on the different phenomena such as inertia, viscosity, conductive heat transport, and diffusive mass. These groups have also been used for predicting wear in polymers and their composites [35], for analyzing lubrication and wear effects via the single-point incremental sheet forming process using the Stribeck curve [36], in correlating wave properties in critical heat transfer transition [37], fan modeling and design [38], the electrohydrodynamics process [39], and even to optimize human energy consumption by using anthropometric, environmental, and workstation variables [40].

In this article, we present a novel approach, aiming to create a unified mathematical model for the entire UMIM process. Unlike previous studies that focused on specific UMIM phenomena and mathematical models, our mathematical approach addresses the gap in the literature by integrating these phenomena into a single analytical framework. Leveraging dimensionless groups, a well-established tool in various fields, we unveiled the intricate interplay of process parameters and material properties in UMIM. This pioneering approach promises valuable insights for optimizing UMIM, enhancing energy efficiency, and improving the mechanical performance of the produced specimens.

## 2. Mathematical Modeling through Dimensionless Groups

### 2.1. Brief Explanation of the Buckingham Pi Theorem

The study of a system consists in establishing relationships between a particular phenomenon and the different parameters that represent the interaction of the system with the environment. The premise of dimensional analysis is that these relationships remain valid regarding the magnitudes of the base units [34].

The basic theorem of dimensional analysis, the Pi theorem, named by P. W. Bridgman from the 1914 theorical work of E. Buckingham [33], allows the derivation of an equation that contains only products of variables with, sometimes, a single non-dimensional product [41,42].

If a physical phenomenon or system has *n* independent variables *a*_1_, *a*_2_, …, *a_n_*, where *k* of them has an independent dimension, and one dependent variable *a*, then *a* is a function of *a*_1_, *a*_2_, …, *a_n_*, so it can be expressed as:

(1) $$a=f\left(a_{1},a_{2},\ldots ,a_{k},a_{k+1},a_{k+2},\ldots ,a_{n}\right)$$

Equation (1) expresses the existence of the relation between the independent variables, which represent the governing parameters of the system, and the dependent variable, and this can be reorganized as:

(2) $$\Pi_{0}=f\left(\Pi_{1},\Pi_{2},\ldots ,\Pi_{k}\right)$$

which contains *n* − *k* dimensional variables, expressed as:

(3) $$\Pi_{1}=\frac{a_{1}}{{a_{1}}^{p_{1}}{a_{1}}^{p_{2}}\ldots {a_{k}}^{p_{k}}},\Pi_{2}=\frac{a_{2}}{{a_{1}}^{q_{1}}{a_{1}}^{q_{2}}\ldots {a_{k}}^{q_{k}}},\ldots ,\Pi_{n-k}=\frac{a_{n}}{{a_{1}}^{r_{1}}{a_{1}}^{r_{2}}\ldots {a_{1}}^{r_{k}}}$$

This Π*_n_* variable is called a Pi group. All the Pi groups in a system are mutually independent, any of the Pi variables can be replaced in combination with other Pi variables, and all of them have a dimension of 1.

(4) $$\Pi_{n}=1$$

Equation (2) is the final result of dimensional analysis, denoting the relationships between a set of physical quantities expressed in dimensionless form, reducing the number of independent variables in Equation (1) from *n* to *n* − *k*. This process simplifies the problem while retaining the functional relationship between the variables [41].

It is of upmost importance to mention that the function obtained in Equation (2) represents a transformation where one determines the number of quantities that affect the dependent variable, not the exact solution to the problem. Neither dimensional analysis nor the Pi theorem are capable of giving an exact functional relationship; therefore, an experimental analysis must be performed. After processing experimental or numerical results, independent variables can be divided into domains, and a power law formula can be adopted having constant values which suit the result in each domain [33], such as:

(5) $$\Pi_{0}=c\cdot \Pi_{1}^{\alpha }\Pi_{2}^{\beta }\ldots \Pi_{n-k}^{\delta }$$

where *c* is a constant, and *α*, *β*, …, *δ* are real numbers. This can also be represented in logarithmic form so that in a double-logarithmic diagram, *α*, *β*, …, *δ* would be the slopes:

(6) $$log\Pi =logc+\alpha log\Pi_{1}+\beta log\Pi_{2}+\cdots +\delta log\Pi_{n-k}$$

### 2.2. Variable Selection

**Figure 2:**
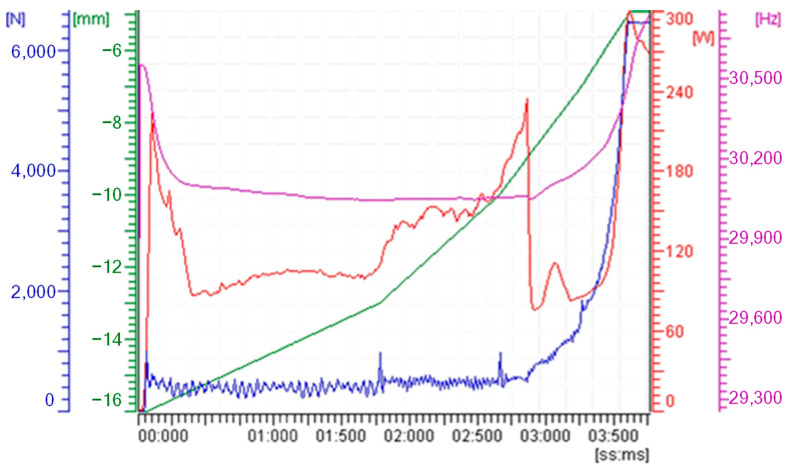
Sonorus 1G processing trends displayed after each injection cycle, monitoring plunger force (blue) and plunger displacement (green), ultrasonic frequency (magenta), and power consumption (red) along the process. The ultrasound energy was computed for 490 J.

Selecting the appropriate variables is crucial for establishing meaningful mathematical relationships using the Buckingham Pi theorem [41]. In our study, all UMIM equipment processing parameters are included in our mathematical model, continuously monitoring, and recording them after each injection cycle (see Figure 2), including energy consumption. These energy consumption data are vital for assessing process stability, which is the primary focus of this research. The equipment’s energy consumption value is derived from the power computed by the ultrasonic generator during a specific injection cycle.

**Figure 3:**
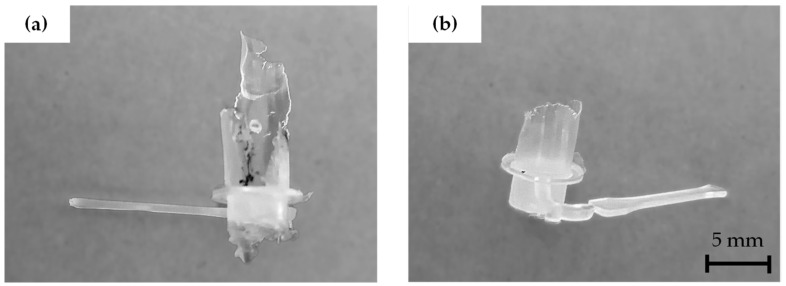
Faulty injections due to molten material flowing over the sonotrode caused by unsuitable combinations of processing parameters, e.g., high injection forces and high vibration amplitude. (**a**) Displays a clear degradation in the overflowed material. (**b**) Injected sample which does not present visible degradation, however, some amount of material flowed upwards to the sonotrode chamber.

Machine power variation is related to the polymer material behavior during ultrasonic injection since inadequately selected processing parameters can cause the molten material to flow upwards, resulting in material degradation. Figure 3 shows faulty injections because of the molten material flowing over the sonotrode. The flowing of molten material over the sonotrode can provoke that the user-specified amplitude not to be reached during the material processing time, requiring the application of more power to keep the ultrasonic parameter values steady.

It is evident that direct responsibility for energy consumption lies with machine parameters such as ultrasonic amplitude, ultrasonic frequency, and ultrasonic time. Some studies found that other machine parameters, such as mold temperature [4,16], injection force [6,13], and injection speed [10,17,19,28,42], have an impact on the processing trends of the machine, leading to the assumption that the full combination of these parameters also affects power requirements. Furthermore, different materials have different responses in the UMIM requiring different combinations of processing conditions, as concluded by Jiang et al. in [13]. They found by using ultrasonic vibrations to molten polymer materials, each material type requires different vibration amplitude, ultrasonic time, and injection force values to be fully plasticized or for avoiding degradation due to each material’s unique properties and characteristics.

One of the main goals is to develop an equation that could describe the material’s response in UMIM, is easy to validate and replicate without extensive material characterization techniques, and that takes into account the polymer properties provided by the technical material datasheets. In this sense, some authors who studied ultrasonic welding [43,44,45] use the dynamic or complex modulus E* of polymers in their formulations:

(7) $$E^{*}=\frac{\sigma }{\varepsilon }$$

with

(8) $$\sigma =\sigma_{0}\sin\left(\omega t+\delta \right)$$

and

(9) $$\varepsilon =\varepsilon_{0}sin(\omega t)$$

where *σ* is the engineering stress, *ε* is the unit strain, ω is the frequency of the cyclic load, and *δ* is the phase angle between the strain and stress responses. The complex modulus E* is also defined as:

(10) $$E^{*}=E^{\prime }+iE^{\prime \prime }$$

where the storage and loss moduli *E*′ and *E*″ are defined, respectively, as:

(11) $$E^{\prime }=\frac{\sigma_{0}}{\varepsilon_{0}}cos\delta$$

(12) $$E^{\prime \prime }=\frac{\sigma_{0}}{\varepsilon_{0}}sin\delta$$

From the properties available in the supplier’s datasheet, the flexural modulus is selected as a variable to describe the mechanical behavior of the material in UMIM. Flexural modulus has the same physical meaning as Young’s modulus since it describes the material’s ability to resist deformation under load in its linear elastic region. Under ideal circumstances, both flexural and tensile modulus are equivalent [46], and they are based on the mechanical properties listed in the datasheets; their values are the closest to the dynamic modulus. Furthermore, the polymer Young’s modulus is dependent on temperature [47] which will be considered using the product of the flexural modulus by the mold temperature as a single variable.

Therefore, the variables that will be selected to derive an expression that describes the material behavior during the UMIM will be: energy consumption (*Q*), final specimen Young’s modulus (*E*), ultrasonic amplitude (*A*), ultrasonic time (*Ut*), injection force (*IF*), injection speed (*IS*), raw material flexural modulus (*S_F_*), mold temperature (*T_M_*), and thermal conductivity coefficient (*λ*). Dimensions and their corresponding SI units of these variables are listed in Table 1. The Young’s modulus of the final specimens is used to describe their ability to support loads within the linear elastic region. The dimensionless model does not explicitly account for plastic deformation during the melting and injection process, as its primary aim is to describe how process parameters influence the part’s mechanical performance. Nevertheless, mold temperature, injection speed, and injection force have a significant impact on material rheology and, consequently, plastic deformation, affecting the final product’s mechanical properties.

### 2.3. Obtention of Dimensionless Groups Using the Buckingham Pi Theorem

Henceforth, one can stablish the functional relationship:

(13) $$f\left(Q,A,Ut,P,S_{F}\cdot T_{M},\lambda ,E\right)=0$$

According to the Buckingham Pi theorem, the number of variables *n* = 7 and the number of dimensions *k* = 4 leads to the obtention of *n* − *k* = 7 − 4 = 3 dimensionless groups or Pi groups:

(14) $$\Pi_{0}=\frac{Q}{Ut\cdot P}$$

(15) $$\Pi_{1}=\frac{A^{4}\cdot S_{f}\cdot T_{M}\cdot \lambda }{Ut\cdot P^{2}}$$

(16) $$\Pi_{2}=\frac{A^{3}\cdot E}{Ut\cdot P}$$

which can be correlated as functional relationships:

(17) $$\frac{Q}{Ut\cdot P}=f\left(\frac{A^{4}\cdot S_{f}\cdot T_{M}\cdot \lambda }{Ut\cdot P^{2}}\right)$$

(18) $$\frac{A^{3}\cdot E}{Ut\cdot P}=f\left(\frac{A^{4}\cdot S_{f}\cdot T_{M}\cdot \lambda }{Ut\cdot P^{2}}\right)$$

Energy consumption and the final specimen’s Young’s modulus can be predicted by stablishing these functional relationships as power law formulas:

(19) $$\frac{Q}{Ut\cdot P}=a{\left(\frac{A^{4}\cdot S_{f}\cdot T_{M}\cdot \lambda }{Ut\cdot P^{2}}\right)}^{\alpha }$$

(20) $$\frac{A^{3}\cdot E}{Ut\cdot P}=b{\left(\frac{A^{4}\cdot S_{f}\cdot T_{M}\cdot \lambda }{Ut\cdot P^{2}}\right)}^{\beta }$$

where *a*, *b*, *α*, and *β* are constants which will be obtained using experimental data.

**Figure 4:**
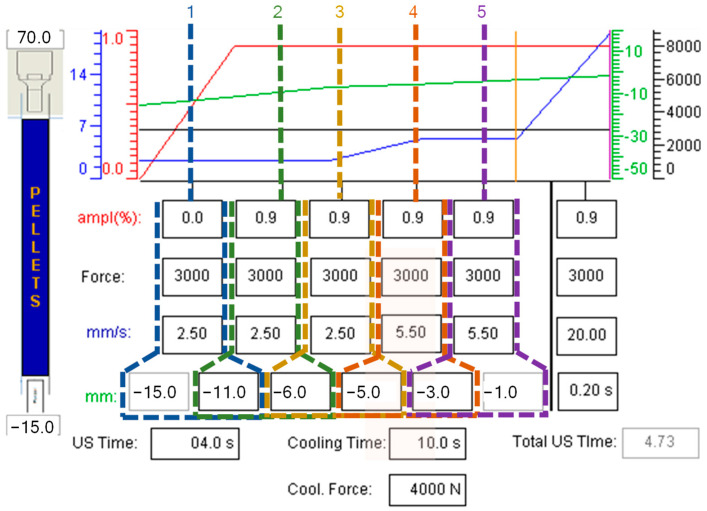
Processing parameters screen on Sonorus 1G. The process is separated into five main stages adjusted by the user, each is established according to the plunger positions, which can move from −23 to −1 mm. At each stage, the plunger force, speed, and amplitude can vary, establishing velocity and force profiles. Above all the adjustable parameters, a graphical representation of the variables along the process is shown.

Since the processing parameters, such as *IS* and *IF*, are important to set the model, it is necessary to investigate how these can be controlled in the injection equipment. Notice from Figure 4 that the equipment allows the control of the injection process dividing the plunger position into five main stages, which can be moved from −23 to −1 mm. At each stage, the user specifies the amplitude percentage, plunger force, plunger velocity or *IS*, and plunger position, which can have different independent values.

Each section has a different length and the plunger is set to traverse each of them at different velocities, for instance, in the first section defined from −15 to −11, the plunger will traverse a length of 4 mm with a velocity of 2.5 mm/s. Following the basic definition of velocity:

(21) $$t=\frac{d}{v}$$

where *v* is the velocity of the plunger, *d* is the length of the section, and *t* the time it takes the plunger to move the length span. The variable *IS* takes the form:

(22) $$IS=\sum_{i=1}^{5}\frac{d_{i}}{t_{i}}$$

Similarly, the variable *IF* is defined as:

(23) $$IF=\sum_{i=1}^{5}{p_{f}}_{i}$$

where *p_f_* is the plunger force at the *i*-th section of the process.

Considering that *IS* and *IF* describe the velocity and the force of a single moving object, the variable plunger power (*P*) is defined in the following form:

(24) P=IF·IS

The validity of proposed dimensionless groups is addressed in Section 4 when compared with experimental data.

## 3. Materials and Methods

### 3.1. Materials

Materials used for this study were different polypropylene (PP) grades, purchased from Indelpro S.A. de C.V. (Altamira, Tam., México). The physical properties are listed in Table 2.

**Figure 5:**
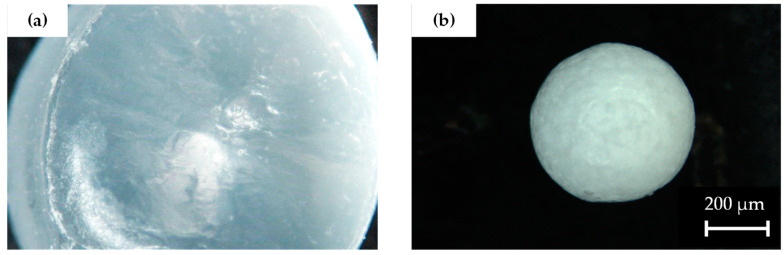
Optical microscopy images of (**a**) PP1 pellet, and (**b**) PP2 pellet.

The PP1 pellets were used as received, while the materials PP2, PP3, and PP4 were sieved with a 1 mm mesh to homogenize pellet size. Figure 5 depicts a visual comparison between PP1 and PP2.

### 3.2. UMIM Equipment

The ultrasonic injection molding machine Sonorus 1G, from Ultrasion S.L. (Barcelona, Spain) was used to produce the micro-tensile specimens. The technical specifications are allocated in Table 3.

### 3.3. Mechanical Properties

**Figure 6:**
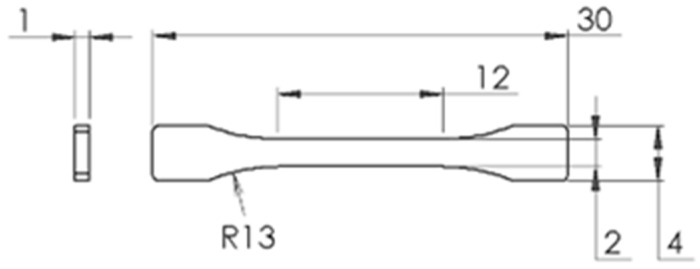
Specimen’s dimensions and geometry, in accordance with ASTM D638-14 [48].

Dog-bone-shaped specimens for tensile tests were fabricated and evaluated according to the ASTM D638-14 standard for tensile properties of plastics [48], with dimensions scaled at 1:5, as illustrated in Figure 6. Mechanical properties of the specimens were found using an Instron 3365 universal testing machine (Norwood, MA, USA) with a load cell of 2.5 kN at room temperature.

### 3.4. Methodology

Information from previous studies [10] was used in conjunction with new experiments in order to obtain a wide variety of measurements. Experiments retrieved from previous studies are labeled as Experiment 1 and 2, where ultrasonic vibration was turned off in the first section of each plunger velocity profile (PVP) for material compaction [16]. *Ut* was set to 4 s and *IF* was constant along the PVP with a value of 3000 N; 15 specimens were produced with each parameter combination. In Experiments 3 to 5, *T_M_* was fixed at 50 °C using a fixed PVP with *IF* constant along it with a value of 6500 N; 10 specimens were produced with each parameter combination. Table 4 includes the PVP used to develop the different Experiments (Exp.) 1–8. The plunger position stages for PVP A were set (−16, −13, −10, −7, −4, and −1) while the others were set (−15, −11, −6.5, −5, −3, and −1).

The results from previous research [10] showed impressive results using PVP B and PP1, therefore, it was used as a pivotal point for the establishment of new ones in Experiments 6 to 8, where 5 specimens were produced with each parameter combination. All parameter combinations used in each Experiment from 1 to 8 are condensed in Table 5.

The material was dried at 80 °C for 6 h in a Heratherm OGS 180 Thermo Scientific oven and stored under vacuum prior to use. In the second experimental set, the pellets were sieved with a 1 mm mesh in order to reduce particle size dispersion. The amount of material per experiment required to fill the plasticizing chamber of the machine depends on the chamber diameter, which is 8 mm, and the plunger’s position specified by the user from −1 to −23 mm into the mold. In Experiments 1, 2, and 6–8, the plunger was placed from position −15 mm to −1 mm, and for Experiments 3–5, from −16 mm to −1 mm.

Tensile tests were carried out as a characterization of the mechanical properties obtained with each combination of parameters of Experiments 6–8.

## 4. Results

### 4.1. Energy Consumption and Mechanical Properties

The average values of energy consumption of the 15 specimens of PP1 produced in Experiments 1 and 2 are shown in Table 6, along with the confidence interval (CI) with a confidence level of 95% (*σ* = 1.96) taking into account the variability in recorded measurements. It can be observed that as the mold temperature increases from 80 to 90 °C the energy consumption exhibits an upward trend, and this behavior is more evidenced when the PVP C is used. When 100% amplitude is applied, the recorded *Q*_avg_ values are higher compared to specimens produced at lower amplitude.

Similarly, Table 7 shows the average energy consumption recorded in Experiments 3–5 to produce 10 specimens using PP2, PP3, and PP4, along with a confidence interval level of 95% (*σ* = 1.96). As the % amplitude and *Ut* rose, the energy consumption values for the different PP grades (i.e., PP2, PP3, and PP4) increased.

In our experimental trials (Exp. 6–8), not all parameter combinations successfully produced complete specimens using PP1. To ensure reliability and consistency, we have included in Table 8 only those combinations that yielded five complete specimens each. This table lists these successfully produced specimens’ average energy consumptions and mechanical properties. Additionally, we calculated the CI at a confidence level of 95% (*σ* = 1.96) to measure the data’s reliability. To assess the mechanical performance of these specimens, we conducted tensile tests, and the maximum average stress and Young’s Modulus values obtained from these tests are also listed in Table 8.

**Figure 7:**
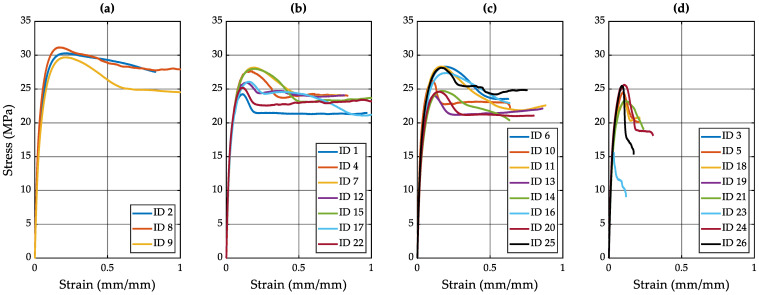
Stress vs. strain curves for specimens produced in Experiments 6 to 8. (**a**) Specimens with ID 2, 8, and 9 exhibit the best structural mechanical properties. (**b**) Specimens with ID 1, 4, 7, 12, 15, 17, and 22 exhibit good load capacity per unit area. (**c**) Decreased ductility properties in specimens with ID 6, 10, 11, 13, 14, 16, 20, and 25. (**d**) Specimens with ID 3, 5, 18, 19, 21, 23, 24, and 26 exhibit decreasing load capacity per unit area and reduced ductility properties.

Figure 7 complements our data by visualizing the stress versus strain experimental curves collected from Experiments 6–8. In Figure 7a, we can observe that specimens with IDs 2, 8, and 9 exhibited the highest uniaxial stress values, which were approximately 30 MPa. Conversely, Figure 7b highlights those specimens with IDs 1, 4, 7, 12, 15, 17, and 22 achieved the greatest elongation values. Figure 7c displays specimens with moderate mechanical properties. However, Figure 7d illustrates that certain specimens exhibited a brittle material behavior, possibly indicating the presence of superficial or molecular defects, such as porosity. It is worth noting that the best mechanical performance was observed in specimens produced under specific conditions: *Ut* = 4 s, *IF* not exceeding 4000 N, *T_M_* = 80 °C, *IS* = 2.9167 mm/s, and *A* = 80% with the PVP B.

### 4.2. Validation of Functional Relationships for Energy Consumption

**Figure 8:**
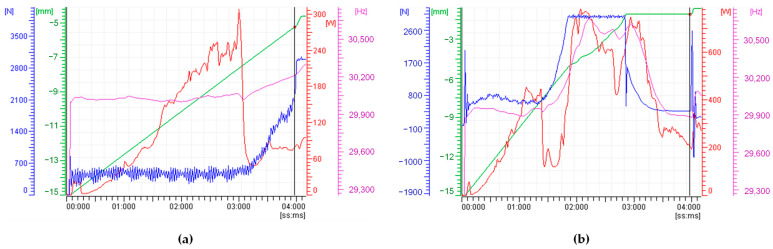
Processing parameters obtained considering different PVP and *Ut* = 4 s. (**a**) PVP B: *IS* = 2.92 mm/s, *Q* = 443 J; (**b**) PVP C: *IS* = 5.5 mm/s, *Q* = 1541 J. Notice that for PVP C the plunger reaches its maximum stroke, and the mold is filled in about 3 s. The additional second of ultrasonic energy that is injected into the specimen might cause some material degradation, as found in [10].

In Figure 8, we explore the energy consumption during the specimen fabrication process, specifically when *Ut* = 4 s. Figure 8b highlights a substantial increase in energy consumption when PVP C is utilized compared to PVP B (see Figure 8a). The energy consumption nearly triples, reaching a total value of 1541 J.

It is worth noting that for PVP C, the plunger reaches its maximum stroke and fills the mold in approximately 3 s. The additional ultrasonic energy injected into the specimen may lead to material degradation, as previously observed in [10]. Therefore, the importance of appropriately tuning injection time and speed becomes evident. This adjustment is crucial for ensuring an efficient production process, preventing unnecessary ultrasonic time that could increase energy consumption and degrade the quality of the specimen material.

**Figure 9:**
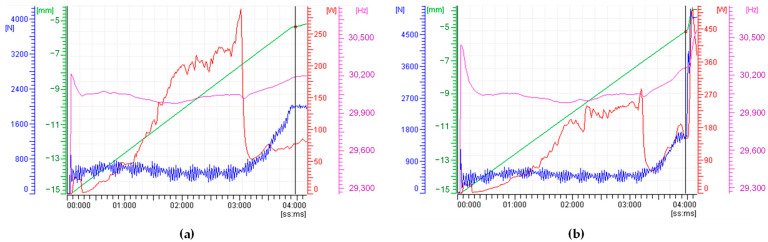
Processing parameters trends of different experimental sets with different *IF* values. (**a**) *IF* = 2000 N; (**b**) *IF* = 5000 N. High injection forces lead to sonotrode overload. Power and frequency peaks can be seen at the end of the micro-injection process. These peaks have higher magnitude values for increasing *IF* values.

Figure 9 illustrates the processing trends of two specimens, produced with identical parameters but varying injection forces. It is noteworthy from Figure 9 that as the injection force increases, there is a corresponding increase in power consumption toward the end of the UMIM process, as indicated by the red line. This phenomenon leads to material overflow along the sonotrode, altering its dynamic performance. Since such a situation is undesirable, one needs to adjust the injection force magnitude.

Furthermore, it is evident that the sonotrode frequency, depicted by the purple line, remains close to 30,200 Hz when an injection force of 2000 N is applied (see Figure 9a). This consistent frequency profile significantly contributes to the production of four full specimens in just five runs. However, this is not the case when injection force values of 3000 N, 4000 N, and 5000 N are employed. In these cases, the corresponding frequency profile, shown in Figure 9b, exceeds the frequency value of 30,200 Hz to the end of the injection process, slightly exceeding the operational sonotrode longitudinal frequency, having a direct impact on the specimen manufacturing process.

To obtain at least four full specimens, an average of eight machine runs were needed; these results confirm the influence that the injection force magnitude has in the production of full specimens by the ultrasonic micro-injection process.

**Figure 10:**
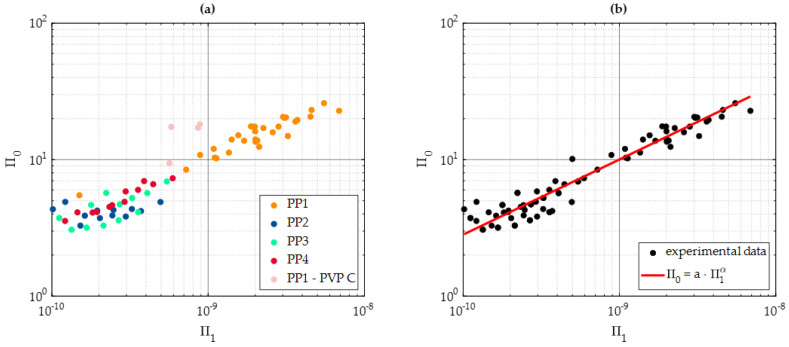
$\Pi_{0}-\Pi_{1}$ plot in logarithmic scales. (**a**) The creation of an exponential plot where most of the experimental points follow the visible trend verifies the correct relationship between Pi groups. Experimental measurements that used PVP C and PP1 are the exception to the obtained relationship, and therefore, these measurements are highlighted as a separate group. (**b**) Fitted curve for Equation (19) using experimental measurements to obtain the *a* and α values to be used in Equation (25) to predict energy consumption as a function of the processing parameters employed.

With the purpose of evaluating the existence of a meaningful functional relationship between the processing parameters and energy consumption, the dimensionless groups obtained in Section 2.2 were plotted as indicated in Equation (17) with $\Pi_{0}$ plotted in the *y*-axis and $\Pi_{1}$ in the *x*-axis. To plot the points shown in Figure 10a, Equations (14) and (15) were used considering PP1, PP2, PP3, and PP4 processing parameters and energy consumption values with λ=0.19 W/m°K [49].

Figure 10a illustrates a clear trend in the plotted values, indicating the presence of an appropriate functional relationship. Notably, some experimental data points located far from the average trend values correspond to specimens made from PVP C, which indicates higher energy consumption during the specimen manufacturing process.

The lower left corner of Figure 10a displays experimental data points corresponding to specimens made from PP2, PP3, and PP4 materials subjected to an *IF* of 6500 N. In this context, the dimensionless group $\Pi_{1}$ serves as a valuable indicator of the likelihood of successfully producing complete specimens (for $\Pi_{1}$ greater than 10^−9^.).

Based on our experimental trials, it is evident that to achieve the production of full specimens, careful tuning of the plunger power (*P*) is essential. This tuning is required to ensure that the energy generated by the sonotrode is sufficient to produce the necessary thermal energy for melting the polymer pellets.

To find the fitting coefficients values that best describe experimental data using Equation (19), it is necessary to write the equation in the form:

(25) $$Q=Ut\cdot P\cdot a{\left(\frac{A^{4}\cdot S_{f}\cdot T_{M}\cdot \lambda }{Ut\cdot P^{2}}\right)}^{\alpha }$$

then, the values of a and α, listed in Table 9, were determined with a confidence level of 95% (σ=1.96). Thus, Equation (19) becomes

(26) $$Q=9.046e^{5}\left(\frac{A^{2.2024}\cdot Ut^{0.4494}{\left(S_{f}\cdot T_{M}\cdot \lambda \right)}^{0.5506}}{P^{0.1012}}\right)$$

Equation (26) can be used to determine the energy consumption, *Q*, as a function of the processing parameters, as shown in Figure 10b where data are shown simultaneously with the fitted curve.

To illustrate the applicability of our derived Equation (26), let us consider the processing conditions for the last set of specimens listed in Table 8: $A=56.2\;{\times \;10}^{-6}\;m$ (100%), $S_{f}=1.4\;GPa$, $T_{M}=100\;{}^{\circ}C=373.15\;{}^{\circ}K$, and Ut=5 s. For the specimens produced with PVP B, the *IS* equals the value of $IS=2.917\;{\times \;10}^{-3}\;m/s$, with IF=3000 N, and P=8.75 W. If we substitute these values into Equation (26), the predicting energy consumption has the value of Q=740.0833 J, which falls within the range of the CI for recorded energy consumption values obtained from experimental measurements (622.6 J and 767 J).

### 4.3. Validation of Functional Relationships for Young’s Modulus

**Figure 11:**
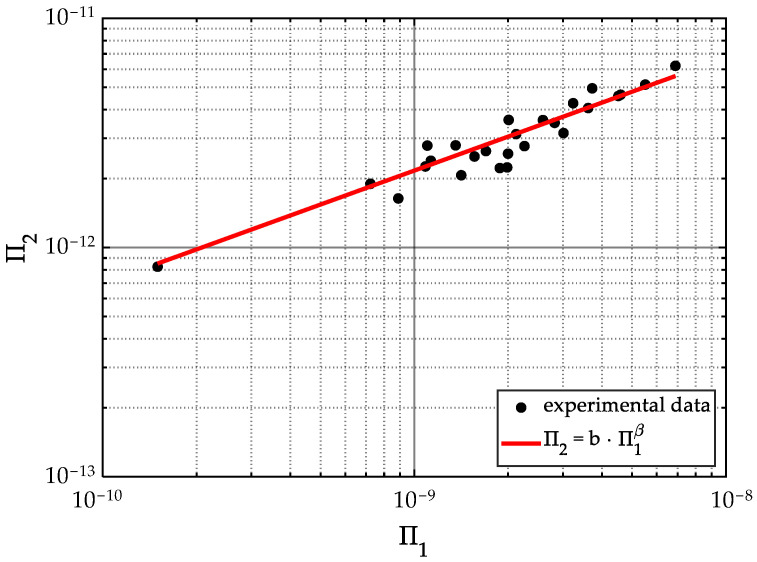
$\Pi_{2}-\Pi_{1}$ plots in logarithmic scales. Notice that most of the experimental points follow a trend which is clearly seen in the logarithmic scale. This confirms the relationship validity between the Pi groups. The fitted curve for Equation (20) using experimental measurements to obtain the *b* and β coefficients values to be used in Equation (27) to predict the Young’s modulus of the final specimens as a function of the processing parameters employed.

With the purpose of investigating a possible relationship between the UMIM processing parameters and the specimen’s Young’s modulus, the dimensionless groups obtained in Section 2.2 are plotted using Equation (20) $\Pi_{0}$ associated with the *y*-axis $\Pi_{2}$ with the *x*-axis. Figure 11 shows the relationship established by Equation (20) λ=0.19 W/m°K [49], and by recalling Equations (14) and (16).

Notice from Figure 11, that the experimental points describe the relationship between dimensionless numbers $\Pi_{1}$ and $\Pi_{2}$ well. Therefore, one can use Equation (20) to predict the material Young’s modulus value. This Equation (20) can be written as:

(27) $$E=\frac{Ut\cdot P}{A^{3}}\cdot b{\left(\frac{A^{4}\cdot S_{f}\cdot T_{M}\cdot \lambda }{Ut\cdot P^{2}}\right)}^{\beta }$$

where the values of the coefficients b and β, with a confidence level of 95% (σ=1.96), are listed in Table 10 and shown in Figure 11.

Thus, the expression that provides the theoretical values of *E* is given as:

(28) $$E=5.821e^{-8}\left(\frac{Ut^{0.5079}\cdot P^{0.0158}\cdot {\left(S_{f}\cdot T_{M}\cdot \lambda \right)}^{0.4921}}{A^{1.0316}}\right)$$

To assess the accuracy attained from our derived expression (28), the processing parameters used to produce specimen ID 26 were considered and listed in Table 5 with $A=56.2\;{\times \;10}^{-6}\;m$, $S_{f}=1.4\;GPa$, $T_{M}=100\;{}^{\circ}C=373.15\;{}^{\circ}K$, and Ut=5 s, with the plunger velocity profile, PVP B, which has an $IS=2.917\;{\times \;10}^{-3}\;m/s$, an IF=3000 N, with P=8.75 W. Substitution of these parameters into Equation (28) yields a theoretical Young’s modulus E=853.0379 MPa that is 1% different from the value of 861.35 MPa listed in Table 8, which was obtained from uniaxial experimental tests. Therefore, it can be concluded that the derived expression (Equation (28)) accurately predicts the Young’s modulus of specimens produced with PP1 via the UMIM process.

It is evident that the dimensionless groups $\Pi_{0}$, $\Pi_{1},$ and $\Pi_{2}$ obtained through the proposed methodology can be used to find a relationship between the UMIM process parameters and the material properties. Its accuracy is linked to the correct determination of the fitting parameters *a*, *b*, *α*, and *β*.

In summary, the Buckingham Pi theorem can be used to correlate operating equipment parameters with the process’s physics and the resulting processing outputs, such as energy consumption and material properties like Young’s modulus. One must bear in mind that the derived Equations (26) and (28) can be applied to other polymeric materials by adjusting the fitting parameters value of *a*, *b*, *α*, and *β* to the material UMIM process conditions for producing a complete specimen. This is a step that needs to be followed when using dimensionless groups since different process conditions lead to the creation of charts with curves that describe the corresponding material response behavior. This approach has been recently used to describe with great accuracy other multi-physic processes, like the additive manufacturing selective laser melting process [50,51].

## 5. Conclusions

In this article, we derived a general expression for calculating energy consumption and linking the UMIM process parameters to the resulting Young’s modulus of different grades of PP using Buckingham Pi theorem of dimensional analysis. These expressions reveal the connections and interactions among material properties and UMIM process parameters, represented by dimensionless groups π_0_, π_1_, and π_2_.

Through the analysis of these dimensionless groups, we found that the most influential UMIM parameters on energy consumption are ultrasonic action time and oscillation wave amplitude, while injection force, injection speed, and mold temperature have a significant impact on final mechanical properties.

Our study demonstrated the accuracy of our derived equations in predicting process energy consumption and Young’s modulus of the specimens. The theoretical energy consumption value was within 3.5% of the machine-computed value, and Young’s modulus prediction differed by only 1% from experimental test results. This elucidates the effectiveness of our Buckingham Pi theorem-based mathematical models for predicting energy consumption and specimen properties by optimizing process parameters.

In summary, our main contributions include the development of accurate dimensionless mathematical expressions for modeling the UMIM process, the identification of key UMIM parameters, the establishment of recommended processing ranges using dimensionless groups, and the successful validation of these relationships through experimental data.

## Figures and Tables

**Table 1:** Variables for dimensional analysis with their corresponding dimensions in SI units.

Variable (Parameter Process)	Variable (Material Properties)	Variable Symbol	Dimensions	SI Units
Amplitude		*A*	L	m
Energy consumption		*Q*	$L^{2}T^{-2}M$	J
	Flexural modulus	*S_F_*	$L^{-1}T^{-2}M$	Pa
Injection force		*IF*	$LT^{-2}M$	N
Injection speed		*IS*	$LT^{-1}$	m/s
Mold temperature		*T_M_*	Θ	°K
Plunger power		*P*	$L^{2}T^{-3}M$	W
	Thermal conductivity coefficient	*λ*	$LT^{-3}M\Theta^{-1}$	W/(m·°K)
Ultrasonic time		*Ut*	T	S
	Young’s modulus	*E*	$L^{-1}T^{-2}M$	Pa

**Table 2:** Polypropylene physical properties according to datasheets.

Properties	Polypropylene Homopolymer Axlene12 (PP1)	Polypropylene Homopolymer HG619N (PP2)	Polypropylene Homopolymer HS013 (PP3)	Polypropylene Homopolymer HG613NW (PP4)
Melt flow index (g/10 min)	12	35	11	20
Yield strength (MPa)	33	33	34	36
Yield strain (%)	10	8	10	9
Notched Izod impact at 23 °C (J/m)	33	15.4	26	26
Flexural modulus (MPa)	1400	1700	1420	1558
Density (g/cm^3^)	0.9	0.9	0.9	0.9
Heat deflection temperature at 0.46 MPa (°C)	104	115	94	111

**Table 3:** Sonorus 1G technical specifications.

Technical Specifications			
Ultrasonic frequency	30 kHz	Clamping force	30 kN
Max. ultrasonic amplitude	56.2 μm	Molding pressure	300 bars (approx.)
Power level	1.5 kW	Max. shot volume	1 cm^3^

**Table 4:** Plunger velocity profiles (PVPs) used in each experiment. PVP-A used a different plunger position stages from the PVP-B to PVP-G.

Plunger Velocity Profile	Stage 1	Stage 2	Stage 3	Stage 4	Stage 5	Injection Speed (*IS*) (mm/s)
PVP A	1.8	3.4	5.0	6.0	9.0	3.72
PVP B	2.5	2.5	2.5	5.5	5.5	2.92
PVP C	5.5	5.5	5.5	5.5	5.5	5.50
PVP D	2.5	3.5	4.5	5.5	6.5	3.60
PVP E	2.0	3.0	3.5	5.0	5.5	2.98
PVP F	3.0	3.5	4.0	4.5	5.0	3.65
PVP G	3.5	3.5	3.5	6.5	6.5	4.03

**Table 5:** Processing parameters tested for each experiment (Exp.).

Processing Parameter	Exp. 1	Exp. 2	Exp. 3	Exp. 4	Exp. 5	Exp. 6	Exp. 7	Exp. 8
*A* (%)	90	100	80	90	100	80	90	100
*Ut* (s)	4	4	(3, 4, 5, 6)	(3, 4, 5, 6)	(3, 4, 5, 6)	(4, 5, 6)	(4, 5, 6)	(4, 5, 6)
*T_M_* (°C)	(80, 90)	(80, 90)	50	50	50	(80, 100)	(80, 100)	(80, 100)
PVP	(B, C)	(B, C)	A	A	A	B, D, E, F, G	B, D, E, F, G	B, D, E, F, G
*IF* (kN)	3.0	3.0	6.5	6.5	6.5	(2, 3, 4, 5)	(2, 3, 4, 5)	(2, 3, 4, 5)
Material	PP1	PP1	PP2, PP3, PP4	PP2, PP3, PP4	PP2, PP3, PP4	PP1	PP1	PP1

**Table 6:** Measured energy consumption for specimens produced in Experiments 1 and 2 using PP1. The experiments were conducted under a fixed *Ut* at 4 s and the *IF* at 3000 N.

*A* (%)	PVP	*T_M_* (°C)	*Q*_avg_ PP1 (J)	CI (J)
90	B	80	473.93	(449.8, 498.1)
		90	483.13	(458.0, 508.3)
	C	80	621.00	(549.5, 692.5)
		90	1146.47	(995.5, 1297.4)
100	B	80	709.73	(659.6, 759.8)
		90	712.87	(668.9, 756.8)
	C	80	1129.43	(942.8, 1316.1)
		90	1198.20	(1054.0, 1342.4)

**Table 7:** Measured energy consumption for specimens produced in Experiments 3–5 using PP2, PP3, and PP4. The experiments were performed under a *T_M_* of 50 °C using a fixed PVP, and *IF* constant of 6500 N.

*A* (%)	*Ut* (s)	*Q*_avg_ PP2 (J)	CI (J)	*Q*_avg_ PP3 (J)	CI (J)	*Q*_avg_ PP4 (J)	CI (J)
80	3	336.92	(320.6, 353.3)	270.75	(260.2, 281.3)	415.5	(407.2, 423.8)
	4	396.83	(388.0, 405.7)	318.17	(304.7, 331.6)	307.58	(293.2, 322.0)
	5	497.67	(480.5, 514.8)	593.25	(577.4, 609.1)	371.25	(356.7, 385.8)
	6	516.42	(509.7, 523.1)	629.25	(609.3, 649.2)	542.58	(529.2, 555.9)
90	3	505.5	(495.0, 516.0)	315.75	(304.7, 326.8)	299.5	(286.3, 312.7)
	4	475.58	(468.7, 482.5)	377.75	(366.5, 389.0)	348.08	(337.9, 358.3)
	5	545.42	(537.7, 553.1)	512.42	(502.2, 522.6)	398	(387.2, 408.8)
	6	600.33	(581.4, 619.3)	564.18	(542.2, 586.1)	675.92	(652.1, 699.8)
100	3	530.33	(513.4, 547.3)	355.17	(346.8, 363.5)	502.25	(469.4, 535.1)
	4	639.58	(628.5, 650.7)	407	(395.0, 419.0)	550.92	(543.4, 558.4)
	5	727	(706.1, 747.9)	463.42	(407.1, 519.8)	634.25	(626.9, 641.6)
	6	849.71	(843.0, 856.5)	622	(564.6, 679.4)	683.42	(643.5, 723.4)

**Table 8:** Measured energy consumption, maximum stress, and Young’s modulus of specimens produced in Experiments 6–8 using PP1.

*A* (%)	*T_M_* (°C)	*Ut* (s)	*IF* (N)	*IS* (mm/s)	PVP	*Q_avg_* (J)	CI (J)	Avg. Maximum Stress (MPa)	Avg. Young’s Modulus (MPa)	ID
80	50	4	6500	3.7221	A	531.00	(479.0, 583.0)	23.52	878.47	1
	80	4	2000	2.9167	B	407.60	(381.8, 433.4)	27.80	898.20	2
		5				497.00	(457.0, 537.0)	28.65	888.94	3
		6				613.60	(488.6, 738.6)	26.68	855.62	4
	100	4		4.0316	G	486.80	(449.5, 524.1)	23.87	799.52	5
		6		2.9167	B	611.40	(562.2, 660.6)	28.02	885.46	6
90	80	4	2000	2.9167	B	481.60	(432.1, 531.1)	29.63	826.46	7
			3000			492.00	(452.5. 531.5)	43.26	974.47	8
			4000			477.60	(448.0, 507.2)	30.53	862.62	9
			5000			493.00	(456.3, 529.7)	27.51	854.85	10
		5	2000			554.80	(500.1, 609.5)	28.02	915.17	11
		6				719.80	(680.5, 759.1)	28.85	854.19	12
	100	4	2000	4.0316	G	651.00	(603.9, 698.1)	23.59	821.63	13
		5	3000			654.75	(614.7, 694.8)	24.94	765.29	14
		4	3000	2.9167	B	434.80	(423.6, 446.0)	30.51	891.03	15
				3.6473	F	493.00	(470.2, 515.8)	25.52	954.51	16
		5		2.9167	B	601.60	(596.4, 606.8)	26.10	838.63	17
				3.6473	F	656.40	(583.3, 729.5)	26.92	844.94	18
		6		2.9167	B	737.20	(676.6, 797.8)	23.17	840.22	19
100	80	4	2000	2.9167	B	531.80	(488.7, 574.9)	24.27	815.34	20
		5				756.40	(666.9, 845.9)	24.91	844.19	21
		6				809.00	(457.0, 537.0)	27.72	915.15	22
		4	4000	3.6473	F	603.60	(558.1, 649.1)	17.42	1016.89	23
	100	5	2000			712.80	(616.9, 808.7)	26.01	943.96	24
		4	3000	2.9167	B	521.20	(492.2, 550.2)	28.02	886.63	25
		5				694.80	(622.6, 767.0)	23.64	861.35	26

**Table 9:** Coefficient values of Equation (25).

Coefficient	Value	Confidence Interval, CI (J)
*a*	9.046 × 10^5^	(3.844 × 10^5^, 1.425 × 10^6^)
α	0.5506	(0.5217, 0.5794)

**Table 10:** Coefficient values for Equations (20) and (27).

Coefficient	Value	Confidence Interval, CI (J)
*b*	5.821 × 10^−8^	(−5.322 × 10^−8^, 1.696 × 10^−7^)
β	0.4921	(0.3947, 0.5896)

## Data Availability

The data presented in this study are available on request from the corresponding author.

## References

[1] Peng T., Jiang B., Zou Y. (2019). Study on the mechanism of interfacial friction heating in polymer ultrasonic plasticization injection molding process. Polymers.

[2] Michaeli W., Kamps T., Hopmann C. (2011). Manufacturing of polymer micro parts by ultrasonic plasticization and direct injection. Microsyst. Technol..

[3] Suslick K.S. (2010). The Chemical Effects of Ultrasound. Sci. Am..

[4] Jiang B., Peng H., Wu W., Jia Y., Zhang Y. (2016). Numerical simulation and experimental investigation of the viscoelastic heating mechanism in ultrasonic plasticizing of amorphous polymers for micro injection molding. Polymers.

[5] Elías-Grajeda A., Vázquez-Lepe E., Siller H.R., Perales-Martínez I.A., Reséndiz-Hernández E., Ramírez-Herrera C.A., Olvera-Trejo D., Martínez-Romero O. (2023). Polypropylene-Based Polymer Locking Ligation System Manufacturing by the Ultrasonic Micromolding Process. Polymers.

[6] Jiang B., Zou Y., Wei G., Wu W. (2019). Evolution of interfacial friction angle and contact area of polymer pellets during the initial stage of ultrasonic plasticization. Polymers.

[7] Jiang B., Hu J., Li J. (2012). Ultrasonic plastification speed of polymer and its influencing factors. J. Cent. South Univ..

[8] Dorf T., Ferrer I., Ciurana J. (2019). The effect of weld line on tensile strength of polyphenylsulfone (PPSU) in ultrasonic micro-moulding technology. Int. J. Adv. Manuf. Technol..

[9] Wu W., Pan L., Li B., He X., Jiang B. (2023). Plastic rod as a promising feed material for enhanced performance of ultrasonic plasticization microinjection molding: Plasticization rate, mechanical and thermal properties. Polym. Test..

[10] Gaxiola-Cockburn R., Martínez-Romero O., Elías-Zúñiga A., Olvera-Trejo D., Reséndiz-Hernández J.E., Soria-Hernández C.G. (2020). Investigation of the Mechanical Properties of Parts Fabricated with Ultrasonic Micro Injection Molding Process Using Polypropylene Recycled Material. Polymers.

[11] Gulcur M., Whiteside B.R., Nair K., Babenko M., Coates P.D. Ultrasonic injection moulding of polypropylene and thermal visualisation of the process using a bespoke injection mould tool. Proceedings of the Euspen’s 18th International Conference & Exhibition.

[12] Masato D., Babenko M., Shriky B., Gough T., Lucchetta G., Whiteside B. (2018). Comparison of crystallization characteristics and mechanical properties of polypropylene processed by ultrasound and conventional micro-injection molding. Adv. Manuf. Technol..

[13] Jiang B., Zou Y., Liu T., Wu W. (2019). Characterization of the Fluidity of the Ultrasonic Plasticized Polymer Melt by Spiral Flow Testing under Micro-Scale. Polymers.

[14] Heredia U., Vázquez E., Ferrer I., Rodríguez C.A., Ciurana J. (2017). Feasibility of manufacturing low aspect ratio parts of PLA by ultrasonic moulding technology. Procedia Manuf..

[15] Michaeli W.H.S., Maesing R., Kamps T. Injection moulding—Machine technology. Proceedings of the 24th international IKV Plastics Technology Colloquium.

[16] Sánchez-Sánchez X., Hernández-Avila M., Elizalde L.E., Martínez O., Ferrer I., Elías-Zuñiga A. (2017). Micro injection molding processing of UHMWPE using ultrasonic vibration energy. Mater. Des..

[17] Dorf T., Perkowska K., Janiszewska M., Ferrer I., Ciurana J. (2018). Effect of the main process parameters on the mechanical strength of polyphenylsulfone (PPSU) in ultrasonic micro-moulding process. Ultrason. Sonochem..

[18] Dorf T., Ferrer I., Ciurana J. (2018). Characterizing ultrasonic micro-molding process of polyetheretherketone (PEEK). Polym. Process..

[19] Ding Q., Du M., Liao T., Men Y., Androsch R. (2022). Polymorphic structure in ultrasonic microinjection-molded poly (butylene-2,6-naphthalate). Polymer.

[20] Sánchez-Sánchez X., Elias-Zuñiga A., Hernández-Avila M. (2018). Processing of ultra-high molecular weight polyethylene/graphite composites by ultrasonic injection mouldingmolding: Taguchi optimization. Ultrason. Sonochem..

[21] Olmo C., Amestoy H., Casas M.T., Martínez J.C., Franco L., Sarasua J.-R., Puiggalí J. (2017). Preparation of nanocomposites of poly(ε-caprolactone) and multi-walled carbon nanotubes by ultrasound micro-molding. Influence of nanotubes on melting and crystallization. Polymers.

[22] Planellas M., Sacristán M., Rey L., Olmo C., Aymamí J., Casas M., del Valle L., Franco L., Puiggalí J. (2014). Micro-molding with ultrasonic vibration energy: New method to disperse nanoclays in polymer matrices. Ultrason. Sonochem..

[23] Díaz A., Franco L., Casas M.T., del Valle L.J., Aymamí J., Olmo C. (2014). Preparation of micro-molded exfoliated clay nanocomposites by means of ultrasonic technology. Polym. Res..

[24] Olmo C., Franco L., del Valle L.J., Puiggalí J. (2019). Preparation of Medicated Polylactide Micropieces by Means of Ultrasonic Technology. Appl. Sci..

[25] Benatar A. (1987). Ultrasonic Welding of Advanced Thermoplastic Composites. Ph.D. Thesis.

[26] Wu W., Peng H., Jia Y., Jiang B. (2017). Characteristics and mechanisms of polymer interfacial friction heating in ultrasonic plasticization for micro injection molding. Microsyst. Technol..

[27] Wu W., He C., Qiang Y., Peng H., Zhou M. (2022). Polymer-Metal Interfacial Friction Characteristics under Ultrasonic Plasticizing Conditions: A United-Atom Molecular Dynamic Study. Mol. Sci..

[28] Marhöfer D.M., Tosello G., Hansen H.N., Islam A. Advancements on the simulation of the micro injection molding process. Proceedings of the 10th International Conference on Multi-Material Micro Manufacture.

[29] Grabalosa J., Ferrer I., Elías-Zúñiga A., Ciurana J. (2016). Influence of processing conditions on manufacturing polyamide parts by ultrasonic molding. Mater. Des..

[30] Janer M., Plantà X., Riera D. (2020). Ultrasonic moulding: Current state of the technology. Ultrasonics.

[31] Gülcür M., Brown E., Gough T., Romano J.-M., Penchev P., Dimov S., Whiteside B. (2020). Ultrasonic micromoulding: Process characterization using extensive in-line monitoring for micro-scaled products. J. Manuf. Process..

[32] Clerk-Maxwell J. (1869). Remarks on the Mathematical Classification of Physical Quantities. Proc. Lond. Math. Soc..

[33] Tan Q.-M. (2011). Dimensional Analysis: With Case Studies in Mechanics.

[34] Sonin A.A. (2001). The Physical Basis of Dimensional Analysis.

[35] Abdelbary A. (2014). Prediction of wear in polymers and their composites. Wear of Polymers and Composites.

[36] Diabb J., Rodríguez C.A., Mamidi N., Sandoval J.A., Taha-Tijerina J., Martínez-Romero O., Elías-Zúñiga A. (2017). Study of lubrication and wear in single point incremental sheet forming (SPIF) process using vegetable oil nanolubricants. Wear.

[37] Sakashita H., Tsuruta T., Nagai N., Mori S., Shoji M., Haramura Y., Ohtake H., Liu W., Umekawa H., Koizumi Y. (2017). CHF-Transition Boiling.

[38] Gullberg P., Sengupta R., Horrigan K. (2013). Transient Fan Modelling and Effects of Blade Deformation in a Truck Cooling Fan Installation.

[39] Estrada-Díaz J.A., Elías-Zúñiga A., Martínez-Romero O., Rodríguez-Salinas J., Olvera-Trejo D. (2021). A mathematical dimensionless model for electrohydrodynamics. Results Phys..

[40] Dhutekar P., Mehta G., Modak J., Shelare S., Belkhode P. (2021). Establishment of mathematical model for minimization of human energy in a plastic moulding operation. Mater. Today Proc..

[41] Gibbings J.C. (2011). Dimensional Analysis.

[42] Ferrer I., Vives-Mestres M., Manresa A., Garcia-Romeu M.L. (2018). Replicability of ultrasonic molding for processing thin-wall polystyrene plates with a microchannel. Materials.

[43] Levy A., le Corre S., Poitou A., Soccard E. (2011). Ultrasonic welding of thermoplastic composites: Modeling of the process using time homogenization. Multiscale Comput. Eng..

[44] Zhang Z., Wang X., Luo Y., Zhang Z., Wang L. (2010). Study on heating process of ultrasonic welding for thermoplastics. Thermoplast. Compos. Mater..

[45] Grewell A., Benatar D. (2007). Welding of plastics: Fundamentals and new developments. Polym. Process..

[46] Giles H.F., Wagner J.R., Mount E.M. (2014). Extrusion: The Definitive Processing Guide and Handbook.

[47] Balani K., Verma V., Agarwal A., Narayan R. (2015). Physical, Thermal, and Mechanical Properties of Polymers. Biosurfaces.

[48] (2014). Standard Test Method for Tensile Properties of Plastics.

[49] Olefins I. (2010). Typical Engineering Properties of Polypropylene.

[50] Estrada-Díaz J.A., Elías-Zúñiga A., Martínez-Romero O., Rodríguez-Salinas J., Olvera-Trejo D. (2021). Mathematical Dimensional Model for Predicting Bulk Density of Inconel 718 Parts Produced by Selective Laser Melting. Materials.

[51] Estrada-Díaz J.A., Elías-Zúñiga A., Martínez-Romero O., Rodríguez-Salinas J., Olvera-Trejo D. (2021). Enhanced Mathematical Model for Producing Highly Dense Metallic Components through Selective Laser Melting. Materials.

